# Why Health Services Research Needs Geoinformatics: Rationale and Case Example

**Published:** 2014-12-01

**Authors:** Tracy Onega, Jennifer Alford-Teaster, Steven Andrews, Craig Ganoe, Mike Perez, King David, Xun Shi

**Affiliations:** 1 Department of Community and Family Medicine, Geisel School of Medicine at Dartmouth, Lebanon, NH, USA; 2 Norris Cotton Cancer Center, Geisel School of Medicine at Dartmouth, Lebanon, NH, USA; 3 The Dartmouth Institute for Health Policy and Clinical Practice, Geisel School of Medicine at Dartmouth, Lebanon, NH, USA; 4 Collaboratory for Healthcare and Biomedical Informatics, Geisel School of Medicine at Dartmouth, Hanover, NH, USA; 5 Exaptive, Inc., Cambridge, MA, USA; 6 The Geography Department, Dartmouth College, Hanover, NH, USA

## Introduction

Delivery of health care in the United States has become increasingly complex over the past 50 years, as health care markets have evolved, technology has diffused, population demographics have shifted, and cultural expectations of health and health care have been transformed. Identifying and understanding important patterns of health care services, accessibility, utilization, and outcomes can best be accomplished by combining data from all of these dimensions in near-real time. The Big Data paradigm provides a new framework to bring together very large volumes of data from a variety of sources and formats, with computing capacity to derive new information, hypotheses, and inferences [1,2]. The complementary fields of genomics and bioinformatics have already made great advances only made possible by Big Data approaches. Similar gains can be made by pairing health services research with geoinformatics –- defined as “the science and technology dealing with the structure and character of spatial information, its capture, its classification and qualification, its storage, processing, portrayal and dissemination, including the infrastructure necessary to secure optimal use of this information” [3]. Integrating geospatial technologies with health services research brings informatics approaches, data sciences, and spatial theories of health and healthcare together to explore relationships among geography, health, and delivery of care in novel ways made possible through geoinformatics. synergy between the two disciplines will enhance our ability to discover how health care is delivered most effectively for the greatest health benefits across populations.

## Shared History of Geography and Health Services Research: Successes and Limitations

Health services research and geography have intersected under the rubrics of medical geography, epidemiology of health care, small area analysis, and public health since ancient Roman times, but more formally since the 1930s. Then, James Glover–an English physician – noticed that tonsillectomies were occurring at highly variable rates across school districts, which could not be explained by geographic, socio-demographic, or clinical factors, and the most likely explanation suggested being differences in how physicians practice [4]. Similar work, begun in the 1970s, became the impetus for the Dartmouth Atlas of Health Care [5], which used health care utilization data to develop geographic units representing health care markets. These health care-based spatial units can be directly compared to identify patterns associated with both effective and ineffective care. Other notable examples of health services research linked to a geospatial framework can be found in the public health arena, with planning of population-based vaccination programs and designation of federally-qualified health centers. Despite these tremendous contributions, limitations exist that geoinformatics and health service research are now poised to overcome as information technology and the digital era expands data availability, accessibility, usability, and timely knowledge generation.

Understanding spatiotemporal distributions of health services is a fundamental aspect of health services research upon which studies of utilization, outcomes, comparative effectiveness, resource allocation, and others are based. Four key limitations can be found in typical approaches to measuring health services distributions: 1. retrospective methods; 2. limited geographic extents; 3. ascertainment challenges; and 4. structured data only; which will be addressed below (Table 1). By combining geoinformatics with health services research, an important domain of questions within the field of medical informatics can be addressed, such as is illustrated with a case example.

## A Health Services Research Problem: Diffusion of Medical Technology

Technological innovation is a hallmark of the U.S. health care system, and relies on the backbone of translational research to evaluate effectiveness after efficacy has been established. The full potential of a new technology is determined as clinical improvements and impacts on population health are assessed. Yet typically diffusion and the research to establish effectiveness occur asynchronously. This limits the potential for timely assessment of broad clinical impact and leads to two concerns: 1. overuse of technologies with unproven or minimal benefits in the general population and concomitant unwarranted costs; 2. underuse of technologies that have beneficial effects and improve outcomes for particular patient populations. As medical technology diffuses into generalizable practice, data and research needs arise to address these concerns from a host of stakeholders perspectives, including patients, clinicians, researchers, health care facilities, commercial vendors, payers, health care systems, communities, and regulatory bodies. A prerequisite for assessing new technologies in community practice is knowledge of the locations or *extent* of diffusion and the populations reached. Data availability and timeliness are critical barriers to establishing this knowledge, contributing to spotty information that is retrospective, often with notable lags. For example, The Dartmouth Atlas of Healthcare [5] and other work [6-8] has described geographic variation of medical technologies at a national level, but relied on Medicare data, which are typically available for research with a lag of 2-3 years. Further, Medicare, as well as private insurers, are dependent on the new technology being approved for reimbursement since they rely on billing data (claims), and coverage of the technology may take years following FDA approval and commercial dissemination, thus not able to be ascertained until unique billing codes are implemented. Some data resources may be timely for monitoring diffusion–such as registries like the Breast Cancer Surveillance Consortium (BCSC) and the HMO Research Network (HMORN) [9,10], and other clinical/provider networks with health information exchanges and/or robust electronic health records. However, these data resources are limited in geographic extent, are often not population-based, and may not capture use of new technologies until they are uniquely coded (e.g. CPT–Common Procedural Terminology), or are reimbursable. Further, most data sources require existing structured data, although natural language processing (NLP) is increasingly applied to unstructured medical record information, which can be powerful, but also time-consuming, complicated and variable across settings [11-13]. Achieving national extent, fully ascertained, and timely data capture to characterize geographic and sociodemographic diffusion of new technologies is a critical need, particularly as the U.S. seeks to improve health and healthcare and limit health care costs to effective use within populations.

## Measuring Near-Real Time Diffusion of Breast Imaging Technology

**Figure 1:**
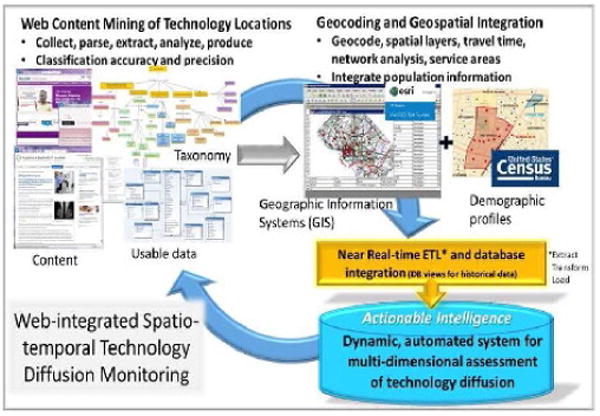
Schematic flow for a geoinformatics application in health services research to dynamically monitor technology diffusion.

To more fully understand dissemination of new technologies–we need to be able to capture the occurrences of the technology in large geographic areas, and in a dynamic way that reflects the dynamic process that dissemination is. Figure 1 presents a schematic approach to do this, using a breast imaging technology diffusion cased example (Figure 1). We can address the existing limitations in measuring, monitoring, and characterizing health care diffusion–with breast imaging as a timely example, given new technologies, such as digital breast tomosynthesis (DBT) [14-16], and legislation related to breast density notification [17]. With a geospatial semantic web [18-22], which combines web mining techniques with geographic information systems and census data, one can ascertain geographic uptake of DBT nationally, estimate potential access overall and by population subgroups, and identify correlates of dissemination patterns.

Web content mining is used in this project to identify instances of DBT based on taxonomy of terms. Using associated web pages from these instances, street address information is captured for the DBT instance. These addresses and related attributes (facility name, date of data capture, etc.) are brought into a GIS for geocoding, spatial joins with other layers (e.g. road network), travel time analysis, and service area creation. Population demographics and other census-derived data are attributed to DBT locations and service areas. This constitutes the database containing geographic extent of DBT diffusion and population characteristics served by DBT facilities, which is refreshed at regular intervals following external validation. This application characterizes heterogeneous diffusion both geographically and socio-demographically, which also serves as a proof of concept for higher-dimension data which incorporates spatial layers, as well as other technologies.

## Conclusion

The application of geoinformatics to health services research has high potential for advancing currently used research methods to monitor and evaluate new technologies as they are translated from experimental settings into communities and populations. A successful application of this approach will yield a validated tool to dynamically integrate geospatial data, population data, and web content for automated discovery and monitoring of technology diffusion. For example, a user interface will provide static functions, such as maps of service locations, derived service areas, density of services available, and populations that are in service catchment areas. Interactive and near-real time functions could produce for user-defined areas, time, and populations: video/time trends of service locations, derive-time defined service areas, time trends of populations coincident with services, and projected population coverage for actual or potential service locations. Such tools can be readily scalable, applicable to other new technologies, and foundational for further capabilities, such as using geostatistical methods for predictive modeling, visualization, and other “Big Data” analytics.

## Figures and Tables

**Table 1:** Summary of major limitations in geographical based health services research and how geoinformatics approaches can be used to address them.

Key Current Limitations in Geographic Based Health Services Research	Example Geoinformatics Approaches to Address Current Limitations
Retrospective methods available data is usually retrospective with lag in timeliness, so pattern described are rarely current.	Using web content mining and mobile technology feeds, near –real time locational data can be obtained.
Limited geographic extents Typically a tradeoff exists between rich/granular data available for small geographic extents, or coarse/ broad data available for large geographic extents.	Use of internet, and mobile- technology based locational data mining, allows for very broad geographic capture, without spatial scale limitations.
Ascertainment Challenges Locational/spatial data is often not available at a point location (address) for health services and/ or patients. Locational data may be only at an area level (e.g. ZIP code), or not known with certainty or completeness at all.	Use of internet, and mobile- technology based locational data mining, is based on either point locations (latitude-longitude) from IP address, or address mining that can be automatically geocoded to point location.
Structured data only typically only spatial data in structured form –such as databases or files-is available. Manual abstraction of spatial data is possible, but limits the scale of examination.	Content Mixing (text and images) allows the use of unstructured data from the internet and mobile technology feeds to Obtain locational information that is not captured explicitly in an existing database.
Asynchronous evaluation of technology with outcomes because evaluation of new technologies is typically limited by the factors above, evaluation of technology occurs *after* it is already being used in actual practice, and often in *small or non representative areas and or populations*. This asynchronicity creates the potential for detrimental outcomes to occur prior to establishing outcomes to be unavailable to populations who may benefit if known that a technology should be available.	By addressing the limitations noted above, evaluation of technology is more likely to occur in near real time, allowing for outcomes to be determined in a more timely manner, rather than with a temporal lag during which patients and populations may be impacted by negative outcomes not previously understood, or missing out on positive outcomes if the technology is reveled to not be located where a population may benefit.
